# Historical Isolation of the Galápagos Carpenter Bee (*Xylocopa darwini*) despite Strong Flight Capability and Ecological Amplitude

**DOI:** 10.1371/journal.pone.0120597

**Published:** 2015-03-25

**Authors:** Pablo Vargas, Beatriz Rumeu, Ruben H. Heleno, Anna Traveset, Manuel Nogales

**Affiliations:** 1 Real Jardín Botánico de Madrid (CSIC-RJB), Madrid, Spain; 2 Department of Life Sciences, Centre for Functional Ecology, University of Coimbra, Coimbra, Portugal; 3 Laboratorio Internacional de Cambio Global (LINC–Global), Institut Mediterrani d’Estudis Avançats (CSIC–UIB), Esporles, Mallorca, Balearic Islands, Spain; 4 Island Ecology and Evolution Research Group, Instituto de Productos Naturales y Agrobiologia (CSIC-IPNA), San Cristóbal de La Laguna, Tenerife, Canary Islands, Spain; Bangor University, UNITED KINGDOM

## Abstract

Colonization across the Galápagos Islands by the carpenter bee (*Xylocopa darwini*) was reconstructed based on distribution of mitochondrial haplotypes (*cytochrome oxidase II* (*COII*) sequences) and haplotype lineages. A total of 12 haplotypes were found in 118 individuals of *X*. *darwini*. Distributional, phylogenetic and phylogeographic analyses suggest early colonization of most islands followed by historical isolation in two main groups: eastern and central-western islands. Evidence of recurrent inter-island colonization of haplotypes is largely lacking, despite strong flight capability and ecological amplitude of the species. Recent palaeogeographic data suggest that several of the current islands were connected in the past and thus the isolation pattern may have been even more pronounced. A contrast analysis was also carried out on 10 animal groups of the Galápagos Islands, and on haplotype colonization of seven animal and plant species from several oceanic archipelagos (the Galápagos, Azores, Canary Islands). New colonization metrics on the number of potential vs. inferred colonization events revealed that the Galápagos carpenter bee shows one of the most significant examples of geographic isolation.

## Introduction

The Galápagos Islands, like many other oceanic archipelagos, are known to have low species diversity, as already acknowledged by Darwin [1]. This sharply contrasts to continental areas on the equator (see [2]). Nevertheless, the Galápagos Islands harbour approximately 2,000 species, of which c. 1,500 are natives (see [3]). Among insect groups, Hymenoptera (with c. 60 species) are clearly underrepresented compared to the species diversity of other insect orders (e.g. Diptera, Lepidoptera). Indeed, Hymenoptera represent a clear example of disharmony of island biota [4]. Only three species of bees, of the around 17,000 species recognised in the world [5], have been reported in the Galápagos Islands, of which the carpenter bee (*Xylocopa darwini* Cockerell, 1926) is found to be the only native (endemic) to the archipelago [6].

The origin of the Galápagos carpenter bee was hypothesized to be associated with an ancestor in the subgenus *Xylocopa* (*Neoxylocopa*), mostly similar to *X*. *carbonaria* [7]. It has been considered that *X*. *darwini* may have been in the archipelago for a relatively long time, although not so long as the other elements of the endemic fauna [8]. Lack of a recent taxonomic account or a phylogenetic reconstruction of *Xylocopa* prevents us to hypothesize closely related species from the Americas. Due to the distance between the Galápagos Islands and the continent (c. 1,000 km), colonization of the islands by *Xylocopa* has primarily been conjectured as via sea dispersal from South America [9]. Further inter-island immigration has been hypothesized to be of sufficient magnitude as to preclude the development of well differentiated races by geographic isolation (see [9]). Therefore, bees appear to have the ability to cross inter-island sea barriers [10]. Since *Xylocopa* bees nest in tree trunks and branches, they may well have reached the islands in driftwood [8]. This hypothesis is supported by the finding of a specific beetle (*Cissites maculata*) that parasites *Xylocopa* species and occurs in the nests of the Galápagos carpenter bee. *Xylocopa*-*Cissites* coexistence has been described as a true phoretic relationship rather than a merely chance occurrence [11,12]. Therefore, the host nest and parasite appear to have been transported and reached the archipelago together. The question remains as to whether recurrent migration among islands has been facilitated by the closer distance between them than that between Galápagos and the mainland. The biology of the Galápagos carpenter bee leads us to interpret high-migration rates favoured by two potential dispersal mechanisms: rafting in driftwood and flying by the adults. Indeed, flying black bees are commonly spotted from boats when several miles away from the nearest coasts [9,10].

Colonization by the carpenter bee may not only have been favoured by these two dispersal mechanisms, but also by the suitability of feeding resources. It is indeed a super-generalist in the plant-pollinator networks of the Galápagos [13,14]. Besides feeding on a high number of plant species, as is characteristic of most *Xylocopa* species, it is present in most terrestrial habitats. However, despite the significant potential for dispersal and establishment, *X*. *darwini* occurs on only 9 of the 12 largest islands [2,3]. The causes of this distribution and the number of inter-island colonizations by *Xylocopa* remain unknown.

The body of knowledge accumulated on the carpenter bee leads us to hypothesize recurrent inter-island colonization due to effective dispersal (nest drift, flight power) and establishment (broad habitat suitability) [15]. Given taxonomic identity, the use of genetic markers is paramount to infer a more realistic number of inter-island colonization events. The distribution of genetic variation across islands helps estimate a higher number of colonizations than that simply inferred from species distribution (i.e. chorology provides the minimum number of colonizations). In particular, haplotype diversity can be used to infer both genotype and lineage connections in a geographic framework by means of a phylogeographic approach [16].

In this study we investigated the colonization history of the carpenter bee as a result of inter-island migration. First, we searched for monophyletic groups and molecular variation across the main islands. Second, island connections were analysed by lineage relationships of genetic (mitochondrial) markers. Finally, we explored patterns of historical isolation and migration based on phylogeographic analyses of insular animals and plants.

## Material and Methods

### Sampling and mitochondrial sequencing

S1 Table shows the list of material sampled from the eight islands where *X*. *darwini* had previously been recorded, plus a new record from the island of Genovesa, a population found during this study and only observed in the last few years (Rosemary Grant, pers. comm.). Baltra and Santa Cruz had a recent land bridge and are thus considered a single island for analysis [17]. Sampling effort was proportional to island size, with seven populations from the island of Isabela (including one from the slopes of each main volcano), and 2–3 from each of the other eight islands (geographic coordinate midpoint: 0° 37´ 90° 21´).

Haplotypes were obtained by sequencing the mitochondrial region of *cytochrome oxidase II* (*COII*) [18]. This region was PCR-amplified with primers E2 (5´ GGCAGAATAAGTGCATTG3´) and H2 (5´CAATATCATTGATGACC3´) using the following conditions: 30 cycles of 94°C for 1 min, 42–50°C for 1 min and 72°C for 1 min, preceded by an initial denaturation at 94°C for 1 min and followed by a final extension at 72°C for 10 min. A volume of 1 μL of bovine serum albumin (BSA) at 1 mg/mL was included in each 25 ml reaction to improve the efficiency of the amplification. PCR products were sequenced using an ABI Prism H 3730xi DNA sequencer at the Macrogen Institute (Korea). Sequences were aligned, and manually adjusted, using MAFFT 6.814b [19] implemented in the Geneious 5.1.7 software [20]. All the new sequences are deposited in the GenBank (see S1 Table for accession numbers).

### Ethics Statement

*Xylocopa darwini* is not a protected species, although occurs in a protected area. The study was approved by the National Park of Galápagos (Ecuador), which provided us the required permits (N°: PC-026-09; N°: PC-04-11).

### Phylogenetic analysis

The main clades of *X*. *darwini* were obtained via Bayesian Inference (BI) analyses. Prior to the Bayesian phylogenetic reconstruction, jModelTest 2.0.2 [21] was used to determine the simplest model of sequence evolution that best fits the sequence data. The BI was implemented in MrBayes 3.1.2 [22] using a HKY+I model for two searches with 10 million generations each and a sample frequency of 1,000. Chain convergence was assessed with Tracer 1.5 [23], and a 50% majority rule consensus tree with Bayesian posterior probabilities (PP) of clades was calculated to obtain the Bayesian estimate of phylogeny after removing the first 25% generations as burn-in.

### Phylogeographic analysis

We inferred connectivity between island populations of *X*. *darwini* through sharing of haplotypes and haplotype lineages. A statistical parsimony method [24] implemented in the TCS 1.21 software [25] was used to infer genealogical relationships among haplotypes. The maximum number of differences resulting from single substitutions among haplotypes was calculated with 95% confidence limits. Two continental *Xylocopa* species (*X*. *(Neoxylocopa)* sp., Puerto López, Manabí, Ecuador; and *X*. *ordinaria*, Colón, Córdoba, Argentina) were used as the outgroup bees to infer ancestral haplotypes.

To reconstruct historical dispersal patterns, a Discrete Phylogeographic Analysis (DPA) was implemented in BEAST 1.6.1 [26] using a standard continuous-time Markov chain as described in Lemey et al. [27]. This analysis determined the probability distribution of several locations (areas) in the nodes of the maximum clade credibility tree. Two approaches were developed, based on geographical distribution: (i) nine areas (nine islands) where *X*. *darwini* is distributed; and (ii) one area per island, plus five more areas (five main volcanoes) of Isabela. Two MCMC analyses were run for 10 million generations, sampling every 1,000th generation. Analysis with Tracer 1.5 [23] confirmed convergence among chains and adequate sample size. Both chains were combined using LogCombiner 1.6.2 after discarding the first 10% of sampled generations as burn-in, and trees were summarized in a maximum clade credibility (MCC) tree obtained in TreeAnotator 1.6.2 and visualized in FigTree 1.4.0. A Bayesian stochastic variable selection model (BSSVS, which is an extension of the discrete phylogeographic model) using the Bayes Factor (BF) test helped identify parsimonious descriptions of the colonization process and achieve statistical significance for the rates of occurrence of dispersal events. The well-supported dispersal rates were visualized in Google Earth (http://earth.google.com) using the tool RateIndicatorBF implemented in BEAST.

### Number of colonization events

A minimum of eight dispersal events are needed to account for inter-island colonization of the carpenter bee based strictly on its distribution (nine islands). Nevertheless, a higher number of colonization events could be interpreted by haplotype distribution (phylogeographic correction). Given that *X*. *darwini* is endemic to Galápagos, we assume that every haplotype originated through a DNA mutation on a single island and then had a similar territory (the number of islands) for dispersal across the archipelago. The haplotypes not involved in a network loop are considered to have a single origin (non-homoplasious) and those forming loops indicate uncertainty in the origin of the nucleotide substitutions (homoplasious) [28]. Accordingly, each non-homoplasious haplotype is interpreted as a unique lineage that could have colonized the nine islands independently. In other words, each haplotype distributed over two or more islands reflects one or more independent colonizations. In contrast, the endemicity of haplotypes on single islands indicates failure in finding molecular evidence for multiple colonization events.

To gain some insight into the colonization success of animal and plant species across oceanic islands, our case study was contrasted with other phylogeographic studies to explore three metrics: (1) minimum number of colonization events taken from chorology (species distribution), i.e. number of islands where the species is present—1; (2) maximum number of colonization events taken from genetics (haplotype number), i.e. number of all haplotypes potentially distributed over all islands -1; and (3) number of inferred colonization events based on actual haplotype distribution, i.e. number of haplotypes present on two or more islands. Finally, an estimate of colonization success is provided by the ratio between (3) and (2), i.e. number of inferred colonization events/ maximum number of potential colonization events.

## Results

### Genetic variation

**Fig 1:**
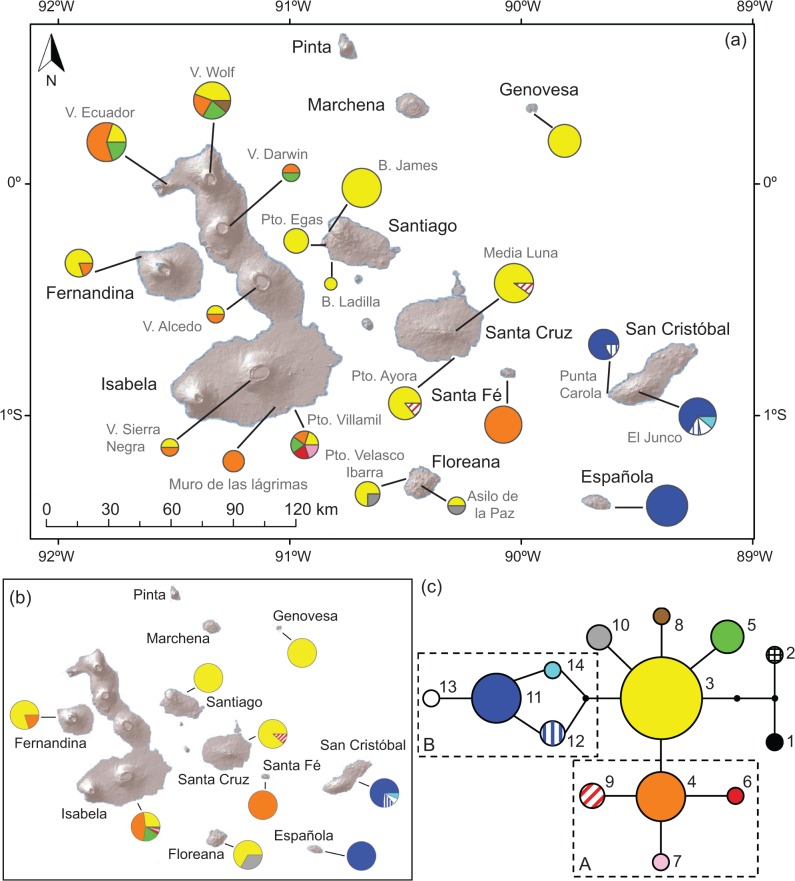
Distribution of genetic diversity (mitochondrial *cytochrome oxidase II* (*COII*) sequences) of *Xylocopa darwini* across the Galápagos Islands. (A) Distribution of mitochondrial haplotypes within populations (see S1 Table). (B) Distribution of haplotypes across islands. (C) Statistical parsimony network of haplotypes; lines represent single nucleotide substitutions, and dots indicate missing haplotypes (extinct or not found). Circle sizes are proportional to the number of sequences obtained for each haplotype.

Sequences were 279 bp long in *X*. *darwini* and displayed no indels. However, a 217 bp fragment was eventually analysed due to low quality of the first 62 bp in many electropherograms. A total of 14 haplotypes were found in 120 *Xylocopa* bees (12 haplotypes in 118 bees of *X*. *darwini*) as a result of 15 nucleotide substitutions. Distribution of haplotypes was imbalanced, with six on Isabela, four on San Cristóbal, two each on Fernandina, Floreana and Santa Cruz, and one on Española, Genovesa, Santa Fe and Santiago (Fig. 1). The highest haplotype diversity within populations was found on Isabela (Puerto Villamil, five haplotypes; Volcán Wolf, four haplotypes) and San Cristóbal (El Junco, four haplotypes) (Fig. 1; see S1 Table). These results indicate that the largest island (Isabela, c. 60% of the Galápagos land area) and the oldest (San Cristóbal, 2.4–4.0 Ma) bear the highest haplotype diversity at both the island and population levels. In addition, single-island (unique) haplotypes were found on Isabela (four haplotypes), San Cristóbal (three), Floreana (one) and Santa Cruz (one).

### Phylogenetic and phylogeographic analyses

The phylogenetic analysis showed a single lineage with a high posterior probability (1 PP), which includes all the samples (26) from the islands of San Cristóbal and Española (S1 Fig.). The haplotype network displayed a high number of tip haplotypes (h5, h6, h7, h8, h9, h10, h13) and only two interior haplotypes (h3, h4). These two interior haplotypes are widely distributed (h3 in 53 samples; h4 in 25 samples), but exclusively found on the western and central islands. Two lineages of four haplotypes each were identified (Fig. 1C). One of them (lineage A) is distributed over central and western islands, whereas the other (lineage B) is located on the two eastern-most islands (San Cristóbal and Española).

**Fig 2:**
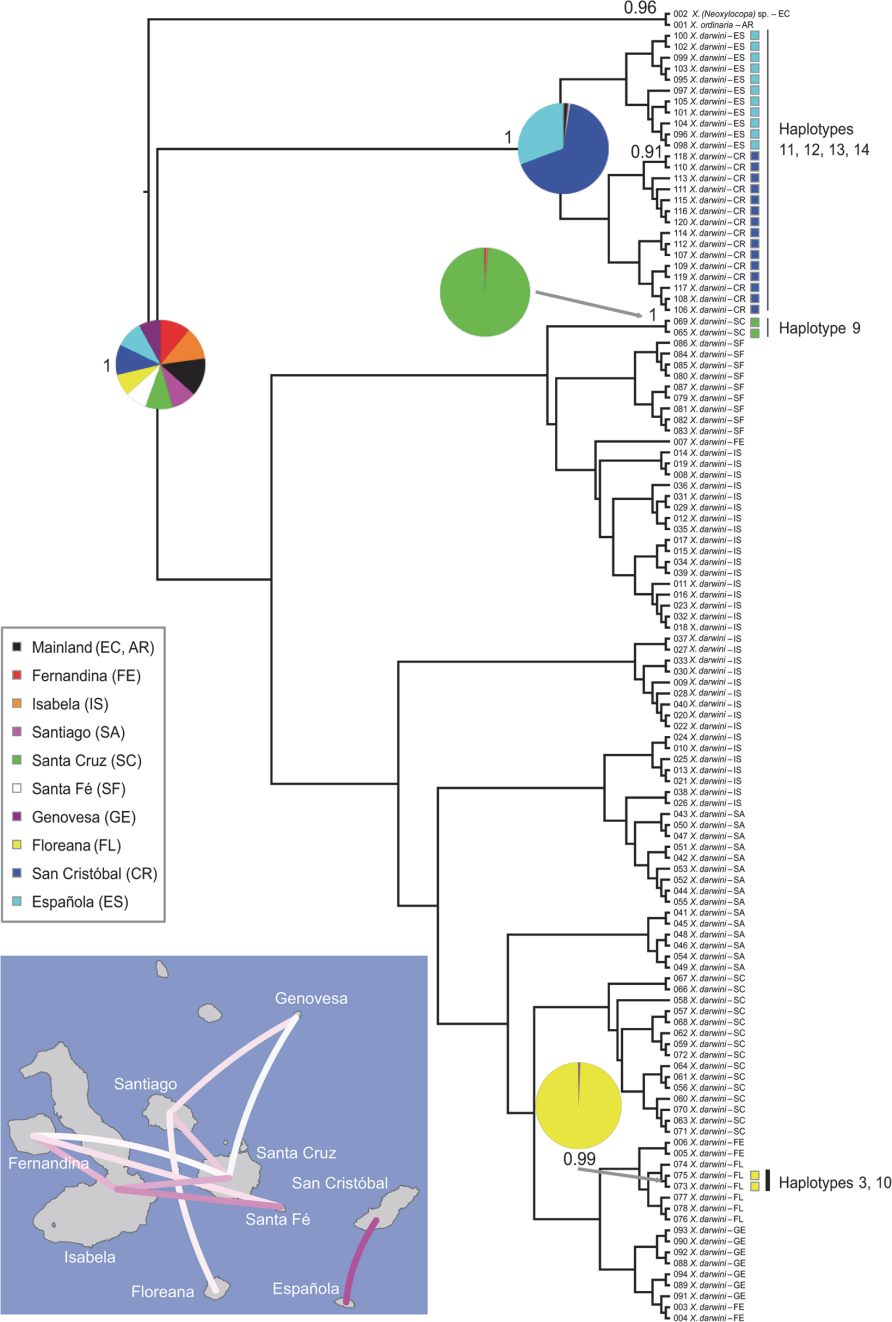
Phylogeographic reconstruction of *Xylocopa darwini*. Maximum clade credibility tree summarized from the geospatial Bayesian analysis of mtDNA (*COII* sequences) of 118 individuals. Pie charts represent posterior probability distributions of the ancestral range at well-supported nodes of interest. Coloured squares represent the sample’s island of origin. Haplotype relatedness is also shown in the well-supported clades. Colonization routes supported by a BF >3 are shown on the map. The colour of each route represents its relative support, with more intense purple lines indicating stronger support.

The Bayesian maximum clade credibility tree based on the DPA of the nine islands showed considerable uncertainty for the geographical origin of *Xylocopa* in the Galápagos Islands (Fig. 2). Similar uncertainty was obtained when dividing the archipelago into 13 areas (considering each volcano of Isabela as a functional “island”) (S2 Fig.). Nevertheless, direct connection between the two continental haplotypes (h1 and h2) and the Galápagos h3 suggests that this interior, widely distributed haplotype is ancestral (Fig. 1). Colonization routes supported by a BF >3 are also shown in Fig. 2. A close connection was found between San Cristóbal and Española and, to a lower extent, between Santiago and Santa Cruz and between some volcanoes of Isabela (S2 Fig.). The only highly supported lineage of more than two samples included all bees from San Cristóbal and Española (Fig. 2). Both analyses support (i) isolation of eastern islands (San Cristóbal and Española), and a close connection between them; and (2) close population relationships among central and western islands.

### Number of haplotypes and colonization events

Inter-island connections can be interpreted exclusively according to the chorological distribution of the species across those islands. In fact, *X*. *darwini* is considered well distributed inasmuch as nine of the 12 islands harbour the bee, and thus a minimum number of eight colonization events can be interpreted. Additional colonization events can be inferred by haplotype distribution. Fig. 1B shows that three haplotypes (h3, h4, h11) are present on more than one island. In particular, the interior h3 is found on six islands, h4 on three and h11 on two. The sharing of two haplotypes between the same two islands indicates one more colonization event connecting them. For example, the fact that Isabela and Fernandina share two haplotypes (h3 and h4), indicates a minimum of two colonization events between these two islands. Isabela shares h3 with Fernadina, Santiago, Santa Cruz, Genovesa and Floreana; and h4 with Fernandina, and Santa Fe. However, only Isabela and Fernandina share more than one haplotype (two). When applying this approach to the other islands, we found that nine haplotypes (h5, h6, h7, h8, h9, h10, h12, h13 and h14) have a single-island distribution, which indicates strong isolation of some lineages of the carpenter bee.

Ocean barriers seem critical to interpret overall isolation by distance in *X*. *darwini*. Isabela and Fernandina are the only islands that share more than one haplotype (two) and are geographically close to each other (c. 4 km apart). In contrast, most haplotypes are broadly spread within each island. For instance, h5 did not succeed in colonizing any island except Isabela, where it spans over 100 km between the southernmost and northernmost populations, a distance longer than the distance between Isabela and nearby islands (Fig. 1A).

### Colonization success of animals and plants

Table 1 shows the metrics used to measure colonization success at the species level. Three animal and four plant species had sufficient sample size and genetic diversity to propose an inferred number of colonization events. Irrespective of the numbers of islands and haplotypes from each study, we could apply our approach to the seven case studies. A higher number of colonization events than that provided by geographic distribution alone was found for each species, except for *Buteo galapagoensis* (see Table 1). Our estimates of colonization success revealed more mobility of plants than that of animals (from high to low): *Olea europaea* subsp. *guanchica* (0.333), *Picconia azorica* (0.250), *Juniperus brevifolia* (0.148), *Cistus monspeliensis* (0.117) and *Buteo galapagoensis* (0.117), *Setophaga petechia* (0.114), *Xylocopa darwini* (0.068). Table 2 summarizes the colonization patterns of 10 different species groups that have been the focus of some phylogenetic studies on oceanic archipelagos.

**Table 1:** Metrics used to infer inter-island colonization success of animal and plant species within the Galápagos, Azores and Canary Islands.

**Species**	**Archipelago (no. individuals sampled)**	^1^ **Number of islands colonized (no. of archipelago islands)**	^2^ **Number of organelle haplotypes**	^3^ **Maximum no. of potential colonization events**	^4^ **Number of inferred colonization events**	^5^ **Colonization success**	**Reference**
*Buteo galapagoensis* (aves)	Galápagos (122)	10 (12)	7	77	9	0.117	[38]
*Cistus monspeliensis* (angiosperms)	Canaries (53)	5 (7)	10	60	7	0.117	[45]
*Juniperus brevifolia* (gymnosperms)	Azores (71)	8 (9)	16	128	19	0.148	[44]
*Olea europaea* subsp. *guanchica* (angiosperms)	Canaries (98)	4 (4)*	11	33	11	0.333	[42]
*Picconia azorica* (angiosperms)	Azores (67)	7 (9)	5	40	10	0.250	[43]
*Setophaga petechia* (aves)	Galápagos (58)	9 (12)	8	88	10	0.114	[40]
*Xylocopa darwini* (Hymenoptera)	Galápagos (118)	9 (12)	12	132	10	0.068	This study

^1^ Number of current islands colonized by each species based on chorological data; (number of archipelago largest islands)

^2^ Number of haplotypes obtained from mitochondria (animals) and plastid (plants) DNA sequences in each study

^3^ Number of haplotypes obtained in each study multiplied by the number of all largest islands-1

^4^ Inter-island colonization events based on distribution of each haplotype, i.e. each colonization event is inferred by haplotype sharing on two or more islands

^5^ Ratio expressing multiple colonization events, that ranges between 1 (all islands colonized by all the haplotypes) and 0 (no inter-island colonization).

* *Olea europaea* subsp. *guanchica* is distributed across the seven Canary Islands. However, the three eastern-most islands show evidence for hybridization with the olive tree. This made the authors use only material from the four western-most islands.

**Table 2:** Patterns of colonization of animal groups (species, genera) across the 12 Galápagos largest islands.

**Species**	**Occurrence (no. islands)**	**Colonization pattern**	**Reference**
*Bulimulus* land snails	7	Early divergence on the southeastern-most islands, followed by northwestern colonization	[34]
*Buteo* hawk	11	Recent arrival (western islands) and rapid population expansion followed by genetic isolation on numerous islands	[38]
*Chelonoidis* tortoises	9	Early divergence on the central and western islands, followed by recurrent inter-island colonization	[17]
*Galagete* moths	12	Radiation related to the chronological emergence of the major islands, followed by extensive colonization to all islands	[39]
*Galapaganus* weevils	9	Early colonization of the young island of Isabela, and notable deviations from the pattern of sequential volcano colonization	[41]
*Microlophus* lizards	12	Early divergence of two lineages primarily distributed in eastern and western islands	[35]
*Mimus* mockingbirds	12	Colonization of more northerly islands from southern islands (Floreana)	[37]
*Phyllodactylus* geckos	10	Ancient colonization before existence of current Galápagos Islands, followed by colonization of central-northern islands and speciation, and an independent colonization from mainland to eastern islands (San Cristóbal)	[36]
*Setophaga* warbler *(S*. *petechia)*	8	Arrival on central islands (S. Cruz, Santiago) followed by colonization of the rest of the Galápagos Islands (and Cocos Island), and medium migration success across islands	[40]
*Xylocopa* bee (*X*. *darwini*)	9	Early arrival on a central-western island, followed by colonization of eastern islands, but low migration among islands afterwards	This study

## Discussion

Colonization of oceanic islands is the result of long-distance dispersal and establishment. It has been historically hypothesized that animals with strong flight capability and broad ecological requirements are better colonizers than those with limited capacity of dispersal and/or establishment [29,30]. Nevertheless, our results based on DNA sequences show that the evolutionary patterns of *X*. *darwini* are complex. Mitochondrial sequence data support a general pattern of historical isolation despite favourable biological characteristics for recurrent colonization across the Galápagos Islands.

### Early colonization of *X*. *darwini*

The phylogenetic and some of the phylogeographic analyses (BI, DPA) did not help finding the closest genetic connection between the mainland and the Galápagos Islands by *Xylocopa*. The network analysis, in turn, identified the interior h3 as the most ancestral genotype (Fig. 1C). This result fits into evolutionary inferences based on the coalescent theory [28]: (i) only three mutation steps separate h3 from the continental haplotypes; (ii) h3 has the highest number (six) of haplotype connections; and (iii) h3 is the most widely distributed (six islands, 53 of 118 bees). Assuming that current geographical distribution of ancestral haplotypes offers a signature of early island colonization, the most ancestral haplotypes (h3, h4) help identify central and western islands of Galápagos as the most likely land for *Xylocopa* arrival. An early colonization of the ancestral h3 is inferred based on its distribution over six of the nine islands. In contrast, the classical hypothesis states that most of the oldest animal lineages in Galápagos occur on the oldest, eastern islands (see references in [31,32], but see Table 2). In particular, the islands of San Cristóbal, Española and Floreana are between 1.5 and 4.0 million years old and harbour many early-diverging lineages of animals. Hence, the colonization sequence of islands for most animals is primarily linked with the volcanic history of the islands. This general pattern has been described for both flightless (see [33] for *Galapaganus* weevils; [34] for Bulimulid land snails; [35] for *Microlophus* lizards; [17] for *Chelonoidis* tortoises; [36] for *Phyllodactylus* geckos) and flying animals (see [37] for *Mimus* mockingbirds; [38] for the *Buteo* hawk; [39] for *Galagete* moths; [40] for the *Setophaga/Dendroica* warbler). However, the historical isolation of the carpenter bee does not fit this general pattern. The two ancestral haplotypes are not present on the eastern islands (San Cristóbal, Española), which leads us to interpret a secondary colonization of these old islands with no further eastern/central-western connection (Fig. 2). In sum, the colonization of the Galápagos Islands by *Xylocopa* shows two spatio-temporal migration episodes: early colonization of central and western islands, followed by a secondary colonization of Española and San Cristóbal, and then differentiation of the two lineages on two groups of islands with no further contact between them.

### Number of colonizations across islands

The study of haplotype distribution across oceanic islands revealed a higher and more realistic number of colonization events than that interpreted exclusively from distributional data (Table 1). Among the species analysed from oceanic archipelagos based on haplotype distribution, the Canarian olive tree (*Olea europaea* subsp. *guanchica*) with 11 colonization events across four islands shows the highest colonization success. Other species groups (Galápagos *Setophaga/Dendroica* warbler, Canarian white-flowered *Cistus*, Azorean juniper, Azorean *Picconia* tree) display lower numbers of colonization events (see Table 1). *Xylocopa* showed one of the strongest patterns of isolation as interpreted by a low number of colonization events (10) across the 12 islands of the Galápagos archipelago. Indeed, many haplotypes (nine) are single-island genotypes, and only a few (three haplotypes) are shared by two or more islands.

This pattern of island isolation is even more pronounced when considering both island proximity and historical land connections in the Galápagos archipelago. Recent research on changes in sea level over the last million years (eustasy) is revealing that six (the western and central islands of Fernandina, Isabela, Santiago, Santa Cruz, Baltra and Santa Fe) of the current 12 large islands were connected around 630 Ka [32]. Accordingly, had *Xylocopa* already inhabited across this ancient macro-island, over-water dispersal among the western-most islands would not be needed to explain distribution of haplotypes 3 and 4 between Isabela and Fernandina (Fig. 1). In fact, many haplotypes from central and western islands could have been extended by terrestrial range expansion. This geological hypothesis would rule out almost completely the role of recurrent colonization of western islands over sea barriers for many organisms (see Table 2), including lack of evidence of colonization across western and central islands, and thus even stronger isolation for *X*. *darwini*.

### Are flying animals more successful in inter-island colonization?

Despite most species of *Xylocopa* being strongly flying bees, migrations between islands by *X*. *darwini* appear to have occurred initially, followed by failure in further colonization. This result is puzzling considering the significant dispersal potential (flight, driftwood) and broad ecological amplitude. Indeed, the carpenter bee feeds on pollen and nectar of flowers from at least 85 of the 403 native species of angiosperms in the Galápagos Islands, and possibly many more, as only a fraction of the native flora has been searched for pollinators [13,14]. Abundance of food resources indicates no trophic limitation for carpenter bee establishment on any islands. In addition, nesting by females in tree wood is not a limitation either since they bore holes about 1 cm deep in branches of the abundant *Bursera graveolens* and *Croton scouleri*, among others [2]. The question remains as to whether other flying animals do not show a predominant pattern of isolation.

The colonization history of some animals is contrasted for some flying and flightless groups across the Galápagos Islands (see Table 2). All of them are distributed over numerous islands, which per se indicate successful colonization. However, some of them show recurrent inter-island colonization, including an interesting range from one of the largest radiations—associated with dispersal across islands in the *Galagete* moths [39]—to the non-speciation and predominant isolation of the Galápagos carpenter bee. Some of the groups presented in Table 2 have a reliably large sample and can be arranged from high-to-low colonization success as follows: *Galagete* moths [39]; *Bulimulus* land snails [34]; *Chelonoides* tortoises [17]; *Galapaganus* weevils [41]; *Setophaga/Dendroica* warbler [40]; *Buteo* hawk [38]; *Microlophus* lizards [35]; *Phyllodactylus* geckos [36]; and *Xylocopa* bee (this study). Surprisingly, no clear pattern of higher colonization by flying animals is therefore observed. When contrasting the colonization success of animals and plants from Galápagos, Azores and Canary Islands simply at the species level, *Xylocopa* shows again the lowest colonization success (from high to low): Canarian olive tree [42]; Azorean *Picconia* tree [43]; Azorean juniper [44]; Galápagos *Buteo* hawk [38] and Canarian white-flowered *Cistus* [45]; Galápagos *Setophaga/Dendroica* warbler [40]; and Galápagos carpenter bee (this study) (Table 1). All these results lead us to conclude that colonization is a highly complex process, and that dispersal capacity (e.g. flight potential) obviously favours but is not sufficient to accurately predict species distributions over time. Indeed, flightless animals (Table 1) and plants with no specific diaspore syndromes for long-distance dispersal [46,47] have successfully colonized most Galápagos islands. To understand these unexpected patterns, the prime role of competition in the establishment process is invoked in some cases (see [48]), In particular, the hypothesis of *competitive exclusion of secondary dispersers*, in which fitness differences would result in failure of secondary colonizations, has gained support to account for isolation patterns of insular animals (see [48]) and plants [44]. Further phylogeographic and ecological studies of floras and faunas will help interpret the importance of competitive exclusion and additional causes involved in the colonization success of oceanic islands.

## Supporting Information

S1 Fig Bayesian Inference (BI) analysis showing main clades of *COII* sequences of *X*. *darwini*. (PDF)

S2 Fig Phylogeographic reconstruction of *Xylocopa darwini* considering each volcano of Isabela as a functional “island”. (PDF)

S1 Table Sampling details of the *Xylocopa* specimens sequenced for the mitochondrial *cytochrome oxidase* II (*COII*). (PDF)
