# Supplementary material for: Historical Isolation of the Galápagos Carpenter Bee (Xylocopa darwini) despite Strong Flight Capability and Ecological Amplitude
Source: PLoS One. 2015 Mar 25;10(3):e0120597. doi: 10.1371/journal.pone.0120597 (PMC4373804; doi:10.1371/journal.pone.0120597)

Historical isolation of the Galápagos carpenter bee (*Xylocopa darwini*) despite strong flight capability and ecological amplitude

Pablo Vargas[1¶], Beatriz Rumeu[1¶], Ruben H. Heleno[2], Anna Traveset[3], Manuel Nogales[4]

Electronic supplementary material (ESM)

Figure S1. Bayesian Inference (BI) analysis showing main clades of *COII* sequences of *X. darwini*. The BI was implemented in MrBayes 3.1.2 [21] using a HKY+I model for two searches with 10 million generations each and a sample frequency of 1,000. Chain convergence was assessed with Tracer 1.5 [22], and a 50% majority rule consensus tree with Bayesian posterior probabilities (PP) of clades was calculated to obtain the Bayesian estimate of phylogeny after removing the first 25% generations as burn-in.

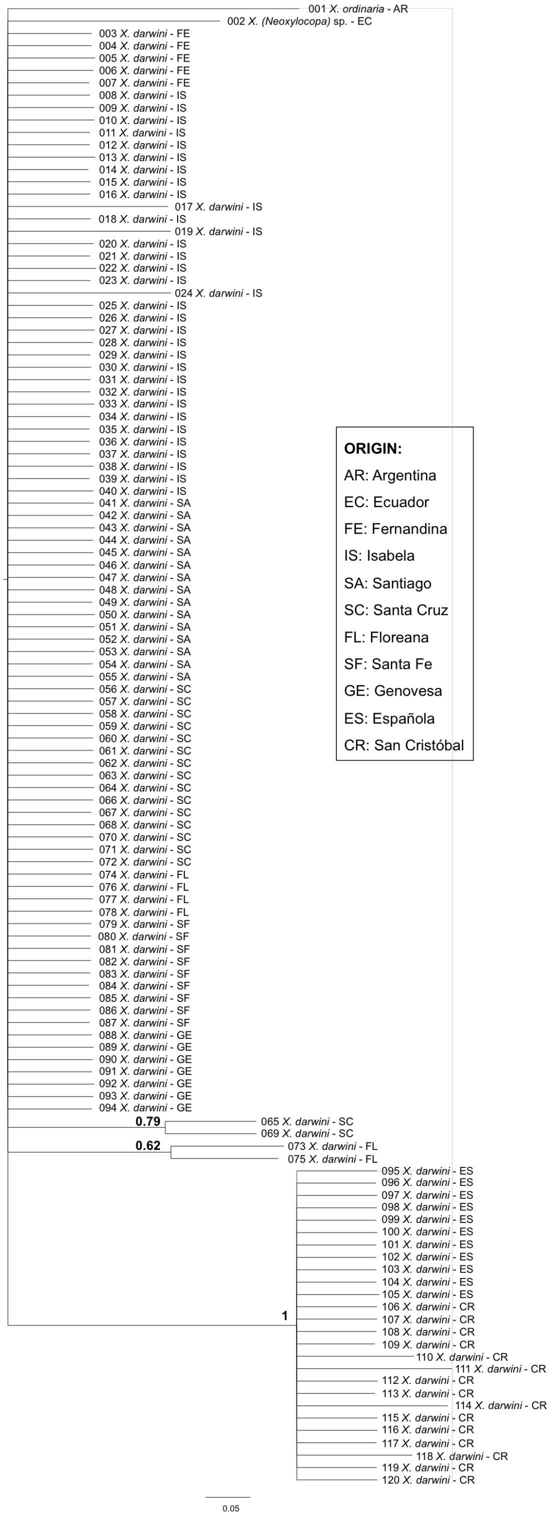

Supplement: S1 Fig — (PDF) [file pone.0120597.s001.pdf]
