# Supplementary material for: Historical Isolation of the Galápagos Carpenter Bee (Xylocopa darwini) despite Strong Flight Capability and Ecological Amplitude
Source: PLoS One. 2015 Mar 25;10(3):e0120597. doi: 10.1371/journal.pone.0120597 (PMC4373804; doi:10.1371/journal.pone.0120597)

Historical isolation of the Galápagos carpenter bee (*Xylocopa darwini*) despite strong flight capability and ecological amplitude

Pablo Vargas[1¶], Beatriz Rumeu[1¶], Ruben H. Heleno[2], Anna Traveset[3], Manuel Nogales[4]

Electronic supplementary material (ESM)

Figure S2. Phylogeographic reconstruction of *Xylocopa darwini* considering each volcano of Isabela as a functional "island". Maximum clade credibility tree summarized from the geospatial Bayesian analysis of mtDNA (*COII* sequences) of 118 individuals. Pie charts represent posterior probability distributions of the ancestral range at well-supported nodes of interest. Coloured rectangles represent the sample's island of origin. Within Isabela, five more areas were analysed as considering main volcanoes: Ecuador, Wolf, Darwin, Alcedo and Sierra Negra. The haplotype relatedness is also shown in the well-supported clades. Colonization routes supported by a BF >3 are shown on the map. The colour of each route represents its relative support, with more intense colours indicating stronger support.

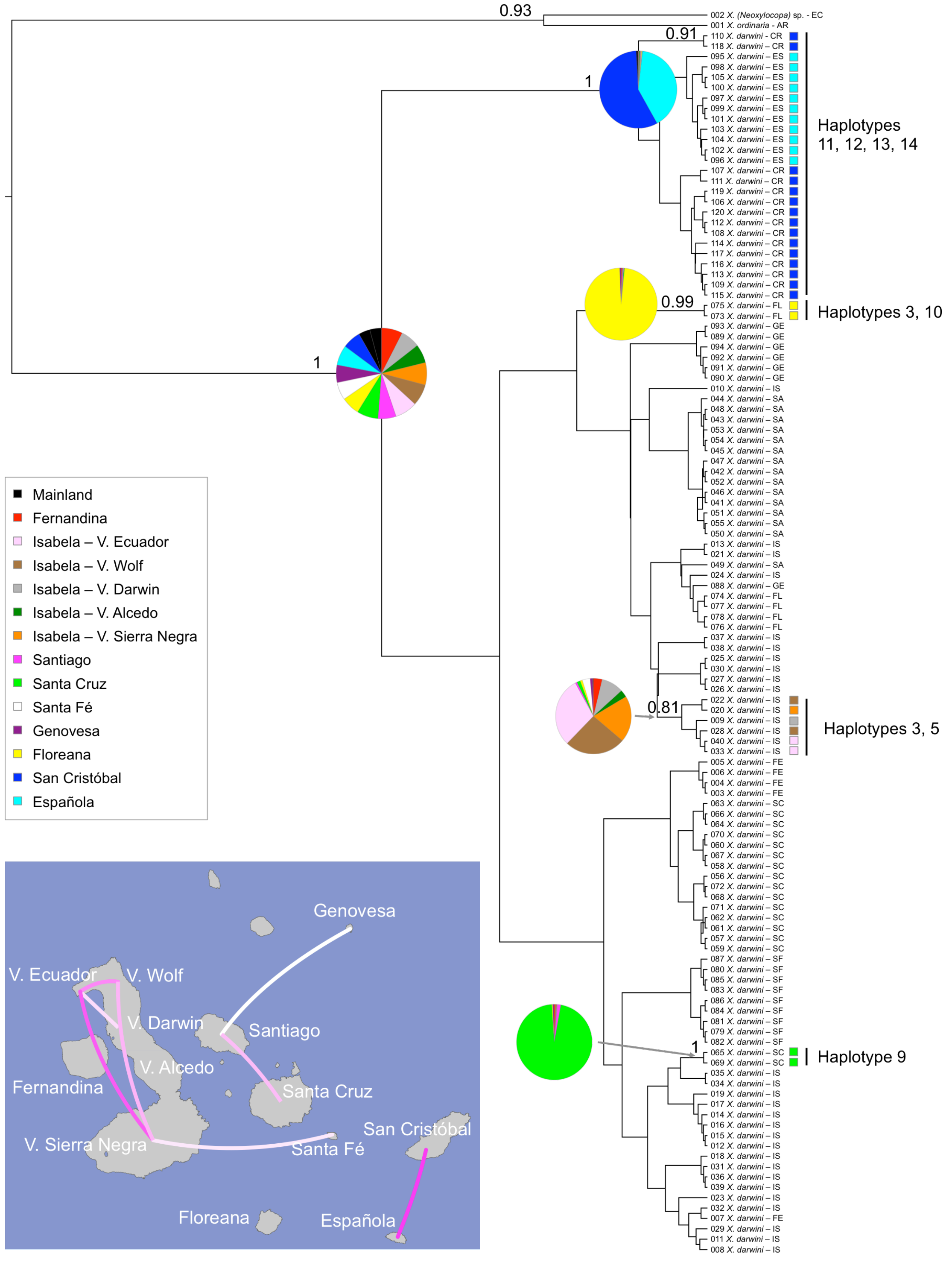

Supplement: S2 Fig — (PDF) [file pone.0120597.s002.pdf]
