# Supplementary material for: Historical Isolation of the Galápagos Carpenter Bee (Xylocopa darwini) despite Strong Flight Capability and Ecological Amplitude
Source: PLoS One. 2015 Mar 25;10(3):e0120597. doi: 10.1371/journal.pone.0120597 (PMC4373804; doi:10.1371/journal.pone.0120597)
Supplement: S1 Table — (PDF) [file pone.0120597.s003.pdf]

Historical isolation of the Galápagos carpenter bee (*Xylocopa darwini*) despite strong flight capability and ecological amplitude

Pablo Vargas[1¶], Beatriz Rumeu[1¶], Ruben H. Heleno[2], Anna Traveset[3], Manuel Nogales[4]

Electronic supplementary material (ESM)

Table S1. Sampling details of the *Xylocopa* specimens sequenced for the mitochondrial *cytochrome oxidase* II (*COII*). Codes as in Figure 2. GenBank accession numbers are shown for each bee. h: haplotypes found according to Figure 1.

No	CODE- voucher	Taxa	ISLAND	Locality	Zone	Plant/habitat where collected	Date	GenBank accession number	h
01	63PV11 (leg. Andrea Coccuci)	*X. ordinaria*		Argentina, Córdoba, Colón	–	–	12.02.2011	KM266466	1
02	15PV11	*X. (Neoxylocopa)* sp.		Ecuador, Guayas, Puerto López	–	–	7.02.2011	KM266467	2
03	21PV10	*X. darwini*	Fernandina	Cabo Douglas	Dry	Flying	11.02.2010	KM266468	3
04	22PV10	*X. darwini*	Fernandina	Cabo Douglas	Dry	*Waltheria ovata*	11.02.2010	KM266469	3
05	24PV10	*X. darwini*	Fernandina	Cabo Douglas	Dry	*Waltheria ovata*	11.02.2010	KM266470	3
06	25PV10	*X. darwini*	Fernandina	Cabo Douglas	Dry	*Waltheria ovata*	12.02.2010	KM266471	3
07	19trisPV11	*X. darwini*	Fernandina	Cabo Douglas	Dry	–	14.02.2011	KM266472	4
08	18PV11(1)	*X. darwini*	Isabela	Playa Tortuga Negra (Volcán Darwin)	Dry	*Lycopersicum*, *Galvezia leucantha*	14.02.2011	KM266473	4
09	18PV11(2)	*X. darwini*	Isabela	Playa Tortuga Negra (Volcán Darwin)	Dry	*Lycopersicum*, *Galvezia leucantha*	14.02.2011	KM266474	5
10	115PV11(1)	*X. darwini*	Isabela	Volcán Alcedo, Pega Pega	Transition	Flying	April - 2001	KM266475	3
11	116PV11	*X. darwini*	Isabela	Volcán Alcedo	Humid	Flying	October-2001	KM266476	4
12	120PV11(1)	*X. darwini*	Isabela	Sierra Negra, agricultural area	Humid	Flying	14.03.2011	KM266477	4
13	120PV11(2)	*X. darwini*	Isabela	Sierra Negra, agricultural area	Humid	Flying	14.03.2011	KM266478	3
14	121PV11(1)	*X. darwini*	Isabela	Sierra Negra, Muro de las Lágrimas	Dry	Flying	March - 2011	KM266479	4
15	121PV11(2)	*X. darwini*	Isabela	Sierra Negra, Muro de las Lágrimas	Dry	Flying	March - 2011	KM266480	4
16	121PV11(3)	*X. darwini*	Isabela	Sierra Negra, Muro de las Lágrimas	Dry	Flying	March - 2011	KM266481	4
17	122PV11(1)	*X. darwini*	Isabela	Sierra Negra, Puerto Villamil	Dry	Flying	15.03.2011	KM266482	6
18	122PV11(2)	*X. darwini*	Isabela	Sierra Negra, Puerto Villamil	Dry	Flying	15.03.2011	KM266483	4
19	122PV11(3)	*X. darwini*	Isabela	Sierra Negra, Puerto Villamil	Dry	Flying	15.03.2011	KM266484	7
20	122PV11(4)	*X. darwini*	Isabela	Sierra Negra, Puerto Villamil	Dry	Flying	15.03.2011	KM266485	5
21	122PV11(5)	*X. darwini*	Isabela	Sierra Negra, Puerto Villamil	Dry	Flying	15.03.2011	KM266486	3
22	123PV11	*X. darwini*	Isabela	Volcán Wolf	30-750m	Flying	14-17.03.2011	KM266487	5

23	123PV11(2)	*X. darwini*	Isabela	Volcán Wolf	30-750m	Flying	14-17.03.2011	KM266488	4
24	123PV11(3)	*X. darwini*	Isabela	Volcán Wolf	30-750m	Flying	14-17.03.2011	KM266489	8
25	123PV11(4)	*X. darwini*	Isabela	Volcán Wolf	30-750m	Flying	14-17.03.2011	KM266490	3
26	123PV11(5)	*X. darwini*	Isabela	Volcán Wolf	30-750m	Flying	14-17.03.2011	KM266491	3
27	123PV11(6)	*X. darwini*	Isabela	Volcán Wolf	30-750m	Flying	14-17.03.2011	KM266492	3
28	123PV11(7)	*X. darwini*	Isabela	Volcán Wolf	30-750m	Flying	14-17.03.2011	KM266493	5
29	123PV11(8)	*X. darwini*	Isabela	Volcán Wolf	30-750m	Flying	14-17.03.2011	KM266494	4
30	123PV11(9)	*X. darwini*	Isabela	Volcán Wolf	30-750m	Flying	14-17.03.2011	KM266495	3
31	124PV11(1)	*X. darwini*	Isabela	Volcán Ecuador	0-550m	Flying	18-19.03.2011	KM266496	4
32	124PV11(2)	*X. darwini*	Isabela	Volcán Ecuador	0-550m	Flying	18-19.03.2011	KM266497	4
33	124PV11(3)	*X. darwini*	Isabela	Volcán Ecuador	0-550m	Flying	18-19.03.2011	KM266498	5
34	124PV11(4)	*X. darwini*	Isabela	Volcán Ecuador	0-550m	Flying	18-19.03.2011	KM266499	4
35	124PV11(5)	*X. darwini*	Isabela	Volcán Ecuador	0-550m	Flying	18-19.03.2011	KM266500	4
36	124PV11(6)	*X. darwini*	Isabela	Volcán Ecuador	0-550m	Flying	18-19.03.2011	KM266501	4
37	124PV11(7)	*X. darwini*	Isabela	Volcán Ecuador	0-550m	Flying	18-19.03.2011	KM266502	3
38	124PV11(8)	*X. darwini*	Isabela	Volcán Ecuador	0-550m	Flying	18-19.03.2011	KM266503	3
39	124PV11(9)	*X. darwini*	Isabela	Volcán Ecuador	0-550m	Flying	18-19.03.2011	KM266504	4
40	124PV11(10)	*X. darwini*	Isabela	Volcán Ecuador	0-550m	Flying	18-19.03.2011	KM266505	5
41	26PV10	*X. darwini*	Santiago	Bahía James, Cerro rojo	Dry	Flying	17.02.2010	KM266506	3
42	26PV10(2)	*X. darwini*	Santiago	Bahía James, Cerro rojo	Dry	Flying	17.02.2010	KM266507	3
43	26PV10(3)	*X. darwini*	Santiago	Bahía James, Cerro rojo	Dry	Flying	17.02.2010	KM266508	3
44	28PV10	*X. darwini*	Santiago	Bahía Ladilla, Cerro colorado	Dry	*Waltheria ovata*	18.02.2010	KM266509	3
45	30PV10	*X. darwini*	Santiago	Bahía James	Dry	Flying	18.02.2010	KM266510	3
46	31PV10	*X. darwini*	Santiago	Bahía James	Dry	*Tamarindus indica*	19.02.2010	KM266511	3
47	31PV10(2)	*X. darwini*	Santiago	Bahía James	Dry	*Tamarindus indica*	19.02.2010	KM266512	3
48	31PV10(3)	*X. darwini*	Santiago	Bahía James	Dry	*Tamarindus indica*	19.02.2010	KM266513	3
49	31PV10(4)	*X. darwini*	Santiago	Bahía James	Dry	*Tamarindus indica*	19.02.2010	KM266514	3
50	31PV10(5)	*X. darwini*	Santiago	Bahía James	Dry	*Tamarindus indica*	19.02.2010	KM266515	3
51	22PV11	*X. darwini*	Santiago	Bahía James, Cerro rojo	Dry	*Galvezia*	19.02.2011	KM266516	3
52	23PV11(1)	*X. darwini*	Santiago	Bahía James – Puerto Egas	Dry	*Cordia leucophlyctis*	19.02.2010	KM266517	3
53	23PV11(2)	*X. darwini*	Santiago	Bahía James – Puerto Egas	Dry	*Cordia psylostachya*	19.02.2010	KM266518	3
54	23PV11(3)	*X. darwini*	Santiago	Bahía James– Puerto Egas	Dry	*Cordia psylostachya*	19.02.2010	KM266519	3
55	23PV11(4)	*X. darwini*	Santiago	Bahía James – Puerto Egas	Dry	*Cordia psylostachya*	19.02.2010	KM266520	3
56	33PV10	*X. darwini*	Santa Cruz	Charles Darwin Foundation (CDF)	Dry	*Tribulus*	20.02.2010	KM266521	3
57	33PV10(2)	*X. darwini*	Santa Cruz	CDF	Dry	*Tribulus*	20.02.2010	KM266522	3
58	34PV10	*X. darwini*	Santa Cruz	Granillo Rojo (Baltra-Puerto Ayora)	Humid	*Scalesia pedunculata, Anoda acerifolia, Alternanthera echinocephala, Sida rhombifolia*	20.02.2010	KM266523	3

59	34PV10(2)	*X. darwini*	Santa Cruz	Granillo Rojo (Baltra-Puerto Ayora)	Humid	*Scalesia pedunculata, Anoda acerifolia, Alternanthera echinocephala, Sida rhombifolia*	20.02.2010	KM266524	3
60	34PV10(3)	*X. darwini*	Santa Cruz	Granillo Rojo (Baltra-Puerto Ayora)	Humid	*Scalesia pedunculata, Anoda acerifolia, Alternanthera echinocephala, Sida rhombifolia*	20.02.2010	KM266525	3
61	34PV10(4)	*X. darwini*	Santa Cruz	Granillo Rojo (Baltra-Puerto Ayora)	Humid	*Scalesia pedunculata, Anoda acerifolia, Alternanthera echinocephala, Sida rhombifolia*	20.02.2010	KM266526	3
62	35PV10	*X. darwini*	Santa Cruz	Media Luna	Humid	*Stachytarpheta cayannensis*	20.02.2010	KM266527	3
63	35PV10(2)	*X. darwini*	Santa Cruz	Media Luna	Humid	*Stachytarpheta cayannensis*	20.02.2010	KM266528	3
64	35PV10(3)	*X. darwini*	Santa Cruz	Media Luna	Humid	*Stachytarpheta cayannensis*	20.02.2010	KM266529	3
65	35PV10(4)	*X. darwini*	Santa Cruz	Media Luna	Humid	*Stachytarpheta cayannensis*	20.02.2010	KM266530	9
66	35PV10(5)	*X. darwini*	Santa Cruz	Media Luna	Humid	*Stachytarpheta cayannensis*	20.02.2010	KM266531	3
67	35PV10(6)	*X. darwini*	Santa Cruz	Media Luna	Humid	*Stachytarpheta cayannensis*	20.02.2010	KM266532	3
68	36PV10	*X. darwini*	Santa Cruz	CDF – Scientific path	Dry	*Lantana peduncularis*	20.02.2010	KM266533	3
69	36PV10(2)	*X. darwini*	Santa Cruz	CDF – Scientific path	Dry	*Lantana peduncularis*	20.02.2010	KM266534	9
70	37PV10	*X. darwini*	Santa Cruz	CDF – Scientific path	Dry	*Tournefortia psilostachya*	20.02.2010	KM266535	3
71	38PV10	*X. darwini*	Santa Cruz	CDF – Scientific path	Dry	*Cordia leucophlyctis*	20.02.2010	KM266536	3
72	114PV11	*X. darwini*	Santa Cruz	CDF, Puerto Ayora	Dry	Flying	09.03.2011	KM266537	3
73	15PV10	*X. darwini*	Floreana	Asilo de la Paz	Humid	*Senna spinosa*?	09.02.2010	KM266538	10
74	16PV10	*X. darwini*	Floreana	Asilo de la Paz	Humid	*Asclepias curasavica*?	09.02.2010	KM266539	3
75	17PV10	*X. darwini*	Floreana	Puerto Velasco Ibarra	Dry	*Cryptocarpus pyriformis*	09.02.2010	KM266540	10
76	18PV10	*X. darwini*	Floreana	Puerto Velasco Ibarra, casa Witmar	Dry	*Delonis regia*	09.02.2010	KM266541	3
77	19PV10	*X. darwini*	Floreana	Puerto Velasco Ibarra, casa Witmar	Dry	Flying	09.02.2010	KM266542	3
78	20PV10	*X. darwini*	Floreana	Puerto Velasco Ibarra, casa Witmar	Dry	*Parkinsonia aculeata*	09.02.2010	KM266543	3
79	9PV12(1)	*X. darwini*	Santa Fé	Bahía de Santa Fé	Dry	Bursera graveolens	16.02.2012	KM266544	4
80	9PV12(2)	*X. darwini*	Santa Fé	Bahía de Santa Fé	Dry	Bursera graveolens	16.02.2012	KM266545	4
81	9PV12(3)	*X. darwini*	Santa Fé	Bahía de Santa Fé	Dry	Bursera graveolens	16.02.2012	KM266546	4
82	9PV12(4)	*X. darwini*	Santa Fé	Bahía de Santa Fé	Dry	Bursera graveolens,	16.02.2012	KM266547	4
83	9PV12(5)	*X. darwini*	Santa Fé	Bahía de Santa Fé	Dry	Bursera graveolens	16.02.2012	KM266548	4
84	9PV12(6)	*X. darwini*	Santa Fé	Bahía de Santa Fé	Dry	*Waltheria ovata*	16.02.2012	KM266549	4
85	9PV12(7)	*X. darwini*	Santa Fé	Bahía de Santa Fé	Dry	*Waltheria ovata*	16.02.2012	KM266550	4
86	9PV12(8)	*X. darwini*	Santa Fé	Bahía de Santa Fé	Dry	*Waltheria ovata*	16.02.2012	KM266551	4
87	9PV12(9)	*X. darwini*	Santa Fé	Bahía de Santa Fé	Dry	*Waltheria ovata*	16.02.2012	KM266552	4

88	4PV12(1)	*X. darwini*	Genovesa	Touristic landing – 2 light houses	Dry	*Opuntia helleri*	13.02.2012	KM266553	3
89	4PV12(2)	*X. darwini*	Genovesa	Touristic landing – 2 light houses	Dry	*Opuntia helleri*	13.02.2012	KM266554	3
90	4PV12(3)	*X. darwini*	Genovesa	Touristic landing – 2 light houses	Dry	*Opuntia helleri*	13.02.2012	KM266555	3
91	4PV12(4)	*X. darwini*	Genovesa	Touristic landing – 2 light houses	Dry	*Opuntia helleri*	13.02.2012	KM266556	3
92	4PV12(5)	*X. darwini*	Genovesa	Touristic landing – 2 light houses	Dry	*Opuntia helleri*	13.02.2012	KM266557	3
93	4PV12(6)	*X. darwini*	Genovesa	Touristic landing – 2 light houses	Dry	*Opuntia helleri*	13.02.2012	KM266558	3
94	4PV12(7)	*X. darwini*	Genovesa	Touristic landing – 2 light houses	Dry	*Opuntia helleri*	13.02.2012	KM266559	3
95	8PV12(1)	*X. darwini*	Española	Playa Manzanillo – Station	Dry	*Waltheria ovata*	15.02.2012	KM266560	11
96	8PV12(2)	*X. darwini*	Española	Playa Manzanillo – Station	Dry	*Waltheria ovata*	15.02.2012	KM266561	11
97	8PV12(3)	*X. darwini*	Española	Playa Manzanillo – Station	Dry	*Waltheria ovata*	15.02.2012	KM266562	11
98	8PV12(4)	*X. darwini*	Española	Playa Manzanillo – Station	Dry	*Waltheria ovata*	15.02.2012	KM266563	11
99	8PV12(5)	*X. darwini*	Española	Playa Manzanillo – Station	Dry	*Waltheria ovata*	15.02.2012	KM266564	11
100	8PV12(6)	*X. darwini*	Española	Playa Manzanillo – Station	Dry	*Waltheria ovata*	15.02.2012	KM266565	11
101	8PV12(7)	*X. darwini*	Española	Playa Manzanillo – Station	Dry	*Waltheria ovata*	15.02.2012	KM266566	11
102	8PV12(8)	*X. darwini*	Española	Playa Manzanillo – Station	Dry	*Waltheria ovata*	15.02.2012	KM266567	11
103	8PV12(9)	*X. darwini*	Española	Playa Manzanillo – Station	Dry	*Waltheria ovata*	15.02.2012	KM266568	11
104	8PV12(10)	*X. darwini*	Española	Playa Manzanillo – Station	Dry	*Waltheria ovata*	15.02.2012	KM266569	11
105	8PV12(11)	*X. darwini*	Española	Playa Manzanillo – Station	Dry	*Waltheria ovata*	15.02.2012	KM266570	11
106	38.1PV10	*X. darwini*	S. Cristóbal	Punta Carola	Dry	Flying	02.03.2010	KM266571	11
107	38.2PV10	*X. darwini*	S. Cristóbal	Punta Carola	Dry	Flying	03.03.3010	KM266572	11
108	38.3PV10	*X. darwini*	S. Cristóbal	Punta Carola	Dry	*Passiflora foetida*	04.03.3010	KM266573	11
109	38.4PV10(1)	*X. darwini*	S. Cristóbal	El Junco	Humid	Flying	05.03.2010	KM266574	11
110	38.4PV10(2)	*X. darwini*	S. Cristóbal	El Junco	Humid	*Cuphea carthaginensis*	05.03.2010	KM266575	12
111	38.4PV10(3)	*X. darwini*	S. Cristóbal	El Junco	Humid	*Cuphea carthaginensis*	05.03.2010	KM266576	13
112	38.4PV10(4)	*X. darwini*	S. Cristóbal	El Junco	Humid	*Cuphea carthaginensis*	05.03.2010	KM266577	11
113	38.5PV10(1)	*X. darwini*	S. Cristóbal	El Junco	Humid	*Cuphea carthaginensis*	05.03.2010	KM266578	11
114	38.5PV10(2)	*X. darwini*	S. Cristóbal	El Junco	Humid	*Polygonum acuminatum*	07.03.2010	KM266579	14
115	38.6PV10	*X. darwini*	S. Cristóbal	El Junco	Humid	*Cuphea carthaginensis*	07.03.2010	KM266580	11
116	38.7PV10(3)	*X. darwini*	S. Cristóbal	Galapaguera	Transition	*Clerodendron molle*	08.03.2010	KM266581	11
117	38.7PV10(4)	*X. darwini*	S. Cristóbal	Galapaguera	Transition	*Cuphea carthaginensis*	08.03.2010	KM266582	11
118	38.8PV10(1)	*X. darwini*	S. Cristóbal	Puerto Baquerizo Moreno	Dry	*Passiflora foetida*	09.03.2010	KM266583	12
119	38.8PV10(2)	*X. darwini*	S. Cristóbal	Puerto Baquerizo Moreno	Dry	*Passiflora foetida*	09.03.2010	KM266584	11
120	119PV11(1)	*X. darwini*	S. Cristóbal	Punta Carola	Dry	Flying	24.03.2011	KM266585	11
